# Impact of Elevated Serum Anti-Müllerian Hormone Levels on Ovulation Induction and Intrauterine Insemination Outcomes in Infertile Women with Polycystic Ovary Syndrome After Failure of ≥3 Clomiphene Citrate Cycles

**DOI:** 10.3390/jcm15062138

**Published:** 2026-03-11

**Authors:** Rifat Taner Aksoy, Gulnur Ozaksit, Gurhan Guney, Meryem Kuru Pekcan, Aytekin Tokmak

**Affiliations:** 1 Department of Reproductive Endocrinology and Infertility, Zekai Tahir Burak Women’s Health Education and Research Hospital, 06230 Ankara, Turkey; muzeyyengulnur.ozaksit@sbu.edu.tr (G.O.); meryem.kurupekcan@sbu.edu.tr (M.K.P.); 2 Department of Reproductive Endocrinology and Infertility, Ankara City Hospital, University of Health Sciences, 06200 Ankara, Turkey; 3 Department of Reproductive Endocrinology and Infertility, Medical Faculty, Balikesir University, 10145 Balikesir, Turkey; gurhanguney@balikesir.edu.tr

**Keywords:** polycystic ovary syndrome, ovulation induction, anti-Mullerian hormone, clomiphene citrate, treatment success

## Abstract

**Background/Objectives:** Although anti-Müllerian hormone (AMH) is a strong biomarker of ovarian reserve and oocyte pools, it is unknown whether high AMH levels can be a reliable predictor of oocyte quality, ovulation, and embryo quality. We aimed to determine whether there is any AMH threshold value that can be used to predict treatment success in women with clomiphene citrate (CC) resistance or failure in polycystic ovary syndrome (PCOS). **Methods:** This retrospective cohort study included 93 infertile women with PCOS who had been previously diagnosed with CC failure or CC resistance between May 2017 and June 2018. Prior to treatment, AMH concentration was measured in all women. The participants were divided into 2 groups according to their conception after ovulation induction (OI) and intrauterine insemination (IUI). At the end of a one-year period, the medical files were assessed retrospectively. Those with and without pregnancy were compared in terms of treatment protocols, infertility periods, laboratory parameters and AMH levels. **Results:** Clinical and biochemical characteristics of 36 pregnant women were compared with those of 57 non-pregnant women. The results showed that the pregnant group had significantly shorter infertility periods and longer ovarian stimulations than the non-pregnant group (*p* < 0.05). Serum AMH levels > 4.5 ng/mL can predict OI and IUI outcome in this specific patient population, with a sensitivity of 56% and a specificity of 69%. Multivariate logistic regression analysis showed that only AMH was identified as an independent predictor of pregnancy [OR = 1.151 (95% CI: 1.034–1.280), *p* = 0.010]. **Conclusions:** Serum AMH may serve as an adjunct predictor of OI and IUI outcomes in infertile women with PCOS who failed to conceive after ≥3 cycles of CC. However, its predictive value appears to be context-dependent and should be interpreted cautiously in clinical practice. Given the distinct clinical characteristics of this patient population, individualized treatment strategies and consideration of earlier alternative therapeutic approaches may be warranted to optimize reproductive outcomes.

## 1. Background

Polycystic ovary syndrome (PCOS) is one of the most common endocrine disorders, affecting 5–10% of women of reproductive age, and is characterized by chronic anovulation, hyperandrogenism, and polycystic ovarian morphology [1,2]. Infertility represents one of the most important clinical consequences of PCOS and primarily develops secondary to ovarian dysfunction-related anovulation [3]. In women with PCOS, the number of preantral and small antral follicles is significantly increased; however, follicular development is arrested at 4–8 mm due to disruptions in the mechanisms regulating normal folliculogenesis. Consequently, dominant follicle selection does not occur, and ovulation fails [4].

Anti-Müllerian hormone (AMH), a member of the transforming growth factor-β (TGF-β) family, is secreted by granulosa cells of preantral and small antral follicles and plays a regulatory role in folliculogenesis. Although AMH is widely accepted as a marker of ovarian reserve, its role in predicting ovulatory function and oocyte quality remains controversial. Women with PCOS typically exhibit serum AMH concentrations two to three times higher than those of women without PCOS, largely due to the increased number of small follicles [5]. In addition to reflecting follicle number, AMH may directly contribute to anovulation in PCOS by inhibiting follicle-stimulating hormone (FSH) sensitivity and aromatase activity, thereby impairing follicular maturation [6]. This pathophysiological role suggests that AMH may not only be a biomarker but also an active participant in ovarian dysfunction.

Clomiphene citrate (CC), a selective estrogen receptor modulator, has long been used as a first-line ovulation induction agent in infertile women with PCOS. By blocking estrogen receptors at the hypothalamic–pituitary level, CC disrupts negative feedback and increases gonadotropin secretion, thereby stimulating follicular development. However, 15–40% of women with PCOS fail to ovulate in response to CC treatment. CC resistance is generally defined as failure to achieve ovulation after at least three cycles of 100–150 mg/day for 5 days, whereas CC failure refers to failure to achieve pregnancy despite ovulation at an optimal dose.

In recent years, several studies have investigated the association between serum AMH levels and clomiphene citrate (CC) resistance in women with PCOS. Most of these studies reported that higher AMH levels are associated with reduced ovulatory response to CC, suggesting that elevated AMH may predict treatment resistance [7,8,9]. However, these studies have important methodological limitations. Many included relatively small sample sizes and were conducted at single centers, limiting generalizability. In addition, different AMH assay methods and varying cut-off values were used. Diagnostic criteria for PCOS and definitions of CC resistance were also inconsistent. Furthermore, potential confounding factors such as BMI, insulin resistance, and androgen levels were not always adequately controlled. As a result, findings have been heterogeneous, and a clear, clinically applicable AMH threshold for predicting CC resistance has not yet been established. Importantly, although previous research has largely focused on the relationship between AMH levels and ovulatory response to CC, and some studies have reported pregnancy outcomes in CC-resistant populations undergoing second-line treatments, the direct effect of baseline AMH levels on pregnancy outcomes in clomiphene-failure infertile women with PCOS has not been specifically evaluated.

Given the psychological burden and economic costs associated with prolonged infertility treatment, the early identification of patients who are unlikely to respond to CC would be clinically valuable. A reliable AMH threshold could help guide individualized treatment strategies, reduce unnecessary delays, and improve overall management outcomes in women with PCOS-related infertility.

Therefore, the present study aimed to evaluate whether serum AMH levels can predict treatment response in women with PCOS exhibiting CC resistance or failure and to determine a clinically relevant AMH threshold. By addressing the methodological limitations of previous research and providing standardized assessment criteria, we aim to contribute more robust evidence to the existing literature and support the development of more precise treatment algorithms for PCOS-associated infertility.

## 2. Material and Methods

### 2.1. Participants

In this retrospective cohort study, 93 infertile women with PCOS who had previously been diagnosed with CC failure or resistance were analyzed at the Zekai Tahir Women’s Health Education and Research Hospital from May 2017 to June 2018.

Infertility was defined as the failure to achieve a clinical pregnancy after 12 months of regular after regular unprotected sexual intercourse. PCOS was diagnosed according to the Rotterdam criteria [10]. The diagnosis required the presence of at least two of the following three features: oligo- or anovulation; clinical and/or biochemical signs of hyperandrogenism; and polycystic ovarian morphology on ultrasonography. Resistance was defined as the failure to achieve ovulation after at least three consecutive cycles of CC administered at the optimal dose while failure was defined as the inability to achieve pregnancy despite ovulation with CC treatment.

Inclusion criteria were women aged 19–35 years with a body mass index (BMI) ≤ 30 kg/m^2^, women with patent tubes on hysterosalpingogram, and partners with normal semen parameters. All patients underwent a 75 g oral glucose tolerance test and showed normal results.

Participants were excluded if they had any endocrine disorder other than PCOS, including thyroid dysfunction, hyperprolactinemia, congenital adrenal hyperplasia, Cushing syndrome, or androgen-secreting tumors. Women with diabetes mellitus, impaired glucose tolerance requiring medical treatment, or those receiving hormonal therapy within the previous three months were also excluded. Those whose medical file data were incomplete or unreliable, and those who were lost to follow-up were also excluded from the study.

Importantly, to minimize potential confounding effects on serum anti-Müllerian hormone (AMH) measurements, women with any acute or chronic systemic disease were excluded from the study. This specifically included autoimmune diseases, chronic inflammatory disorders, infectious diseases, hepatic or renal dysfunction, and any medical condition known to be associated with elevated cytokine levels. Participants with a history of malignancy or those using immunomodulatory or anti-inflammatory medications were also not eligible.

After undergoing OI with intrauterine insemination (IUI), we divided the patients into 2 groups based on whether they became pregnant. At the end of a one-year period, the files were examined retrospectively. Those with and without pregnancy were compared in terms of treatment protocols, infertility periods, laboratory parameters and AMH levels.

### 2.2. Laboratory Studies

Prior to treatment, all cases were assessed for AMH concentration. The demographic and laboratory characteristics of the participants are shown in Table 1. Blood samples were collected on day three after overnight fasting before treatment. Serum samples were separated by centrifugation at 4000 rpm for 10 min and assayed for AMH after collection. An automated electrochemiluminescence immunoassay was used to measure AMH in serum (Elecsys Cobas e 411 analyzer; Roche Diagnostics GmbH, Mannheim, Germany). The AMH concentration range was determined to be 0.01–23 ng/mL, with a minimum detectable AMH dose of 0.01 ng/mL. The intra- and interassay coefficients of variance were <4.4% and <1.8%, respectively. The results are expressed in nanograms per milliliter. A UniCel DxI 800 immunoassay system (Beckman Coulter, Fullerton, CA, USA) was used to measure the serum concentrations of basal hormones, including FSH, luteinizing hormone, estradiol, thyroid-stimulating hormone, and prolactin [11].

### 2.3. Ovulation Induction

All participants were followed for at least three cycles of treatment. We initially administered 100 mg/day CC between days 3 and 5 of the first cycle. If ovulation was not detected, the dose was increased to 150 mg/day in the subsequent cycles. If ovulation occurred, the dose was maintained constant.

The starting dose of 37.5–75 IU/day of FSH (Gonal-F ^®^, Merck Sereno, Aubonne, Switzerland) was started on days 2 or 3 of the cycle in the group receiving Gn therapy if CC treatment was not achieved. A General Electric Medical Systems 7.5 MHz endovaginal probe and Logic 200 Pro ultrasound device were used for all sonographic examinations. A 250 µg dose of recombinant human chorionic gonadotropin (Ovitrelle, Merck Serono, Modugno, BA, Italy) was administered when at least one follicle of 18 mm or larger was observed on transvaginal ultrasound, and IUI was scheduled for 24–36 h later. Although ovulatory dysfunction was the only indication for IUI and semen parameters were within the normal range in all couples, we routinely performed IUI in all participants as a hospital protocol. Semen was collected using the swim-up technique. Sperm analysis was performed by the same andrology laboratory technician according to the hospital quality control program. The IUI procedures were performed with the same type of IUI catheter by clinicians with the same level of experience. Luteal phase support with vaginal progesterone (Progestan^®^ capsule, 200 mg; Kocak Farma, Istanbul, Turkey) was routinely offered immediately after IUI. The drug dosage was individualized according to the patient’s response and/or data from the previous cycles. A qualitative serum beta-human chorionic gonadotropin (beta-hCG) test was performed if menstruation had not started 2 weeks after insemination. Those with BhCG > 10 mIU/mL were considered pregnant.

We also assessed demographic characteristics, such as BMI (kg/m^2^) and age, and clinical factors, such as infertility status (primary or secondary) and cycle characteristics. Participants were stratified into two groups according to pregnancy outcome achieved during subsequent ovulation induction OI and IUI cycles. Patients who fulfilled the predefined diagnostic criteria for CC resistance or CC failure proceeded with OI + IUI using alternative ovarian stimulation protocols during the follow-up period. At the completion of the one-year study period, all medical records were retrospectively reviewed. For women who achieved pregnancy, treatment history was analyzed up to and including the conception cycle. Both prior and current stimulation cycles were recorded in detail. The ovarian stimulation regimens administered during the study period were categorized into three groups: CC + IUI, Gn + IUI, and combined CC + Gn + IUI.

### 2.4. Statistical Analysis

Statistical analysis was performed using the Statistical Package for the Social Sciences (SPSS) version 22.0 (Chicago, IL, USA). The following descriptive statistics were used to describe the study data: mean, median, standard deviation, and frequency. The Shapiro–Wilk test was used to test the distribution of continuous variables. Student’s *t*-test was used for continuous variables with a normal distribution between the groups. In contrast, the Mann–Whitney U test was used for continuous variables that were not normally distributed. Categorical variables were compared between the groups using the chi-square or Fisher’s exact test. The performance of AMH concentration in predicting OI and IUI success in PCOS patients was assessed using receiver-operating characteristic (ROC) analysis. Multivariate logistic regression of baseline risk factors was also used to predict pregnancy in this study population. Statistical significance was set at *p* < 0.05.

## 3. Results

The demographic, clinical, and laboratory characteristics of the study population are summarized in Table 1. A total of 93 women were included in the analysis: 36 who achieved pregnancy following OI + IUI treatment and 57 who did not. The two groups were comparable in terms of age and BMI. There were no significant differences between the pregnant and non-pregnant groups regarding age, BMI, gravidity, parity, abortion history, or baseline hormonal parameters, including follicle-stimulating hormone (FSH), luteinizing hormone (LH), thyroid-stimulating hormone (TSH), estradiol (E2), and prolactin (all *p* > 0.05).

Previous and current ovarian stimulation protocols were similarly distributed between the groups, with no statistically significant differences observed. Endometrial thickness measured on the day of trigger was also comparable between the two groups (*p* > 0.05).

Notably, the duration of infertility was significantly shorter in women who achieved pregnancy compared to those who did not (*p* = 0.008). In addition, the duration of ovarian stimulation was significantly longer in the pregnant group (*p* = 0.028). Serum AMH levels were significantly higher in the non-pregnant group than in the pregnant group (*p* = 0.026).

There were 10 patients (10.8%) with CC resistance and 83 patients (89.2%) with CC failure. There was no statistically significant difference between the pregnant and non-pregnant groups in terms of the number of patients with CC resistance or treatment failure (*p* = 1.000). The mean AMH level was 5.01 ± 3.92 in the CC resistance patients, with a median of 4.16 (min–max: 1.00–26.19) whereas the mean AMH level was 9.67 ± 11.67, with a median of 3.47 (min–max: 1.69–31.71) (*p* = 0.941) in CC failure group.

**Figure 1:**
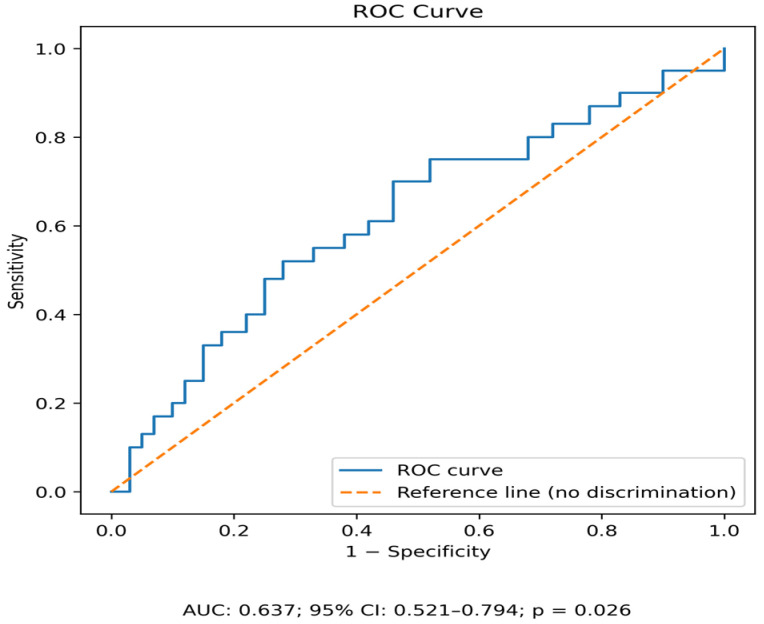
ROC curve analysis for AMH in predicting OI + IUI outcomes.

In the receiver operating characteristic (ROC) curve analysis shown in Figure 1, we found that serum AMH levels higher than 4.5 ng/mL may adversely affect OI + IUI outcomes (pregnancy) in infertile women with PCOS and a history of CC resistance of failure, and the results showed a sensitivity of 57% and a specificity of 68% (Figure 1).

In the multivariate logistic regression analysis, only AMH was identified as an independent predictor of pregnancy (B = 0.140, OR = 1.151, 95% CI: 1.034–1.280, *p* = 0.010). Age, BMI, and duration of infertility were not significantly associated with pregnancy outcomes (*p* > 0.05 for all) (Table 2).

## 4. Discussion

In this study, we investigated the discriminative value of serum AMH levels for pregnancy in infertile women with PCOS with CC resistance or failure. AMH levels were higher in non-pregnant women with PCOS than in pregnant women with PCOS. Our findings demonstrate that women who failed to achieve pregnancy following OI and IUI had significantly higher serum AMH levels compared with those who conceived. ROC curve analysis identified an AMH threshold of ≥4.5 ng/mL as the optimal cut-off value for predicting unsuccessful outcomes following OI and IUI in this specific infertile population. There were no differences in baseline hormone levels or past/current treatment protocols between the two groups.

AMH is well recognized as a marker of ovarian reserve; however, its role in follicular dynamics and ovulatory dysfunction in PCOS is complex. Elevated AMH levels in PCOS are thought to reflect an increased pool of small antral follicles and impaired follicular selection. Experimental and clinical studies have demonstrated that AMH inhibits follicle-stimulating hormone (FSH) action on granulosa cells, thereby suppressing aromatase activity and estradiol production. This inhibitory effect may contribute to CC resistance by preventing adequate follicular recruitment despite increased endogenous FSH secretion induced by CC.

Previous studies have reported a wide range of AMH cutoff values for predicting CC resistance, ranging from 3.4 ng/mL to over 10 ng/mL [12,13]. These discrepancies may be attributed to differences in assay methods, ethnic backgrounds, metabolic profiles, BMI, and study designs. Unlike some previous studies, our cohort consisted of relatively young, non-obese women with normal glucose metabolism, which may explain the moderate cutoff value identified in our analysis. Additionally, all AMH measurements were performed using the same assay under standardized laboratory conditions, enhancing the internal validity of our findings.

Another study evaluating 207 young Korean women reported relatively higher AMH levels and identified a cut-off value of 10.0 ng/mL to distinguish PCOS cases from women without PCOS despite the presence of polycystic ovarian morphology [14]. However, we believe that this high AMH level may not accurately reflect CC resistance. In this study, the researchers claimed that their high AMH levels could be due to higher BMI and long sample storage conditions of up to 30 weeks. In relation to AMH, storage temperature is as important as the storage time. Low freezing temperatures can cause denaturation of AMH protein, resulting in increased AMH levels. In addition, if cytokines are present in the serum, AMH levels may interact with them and affect the measured AMH levels. To minimize potential pre-analytical variability, we excluded participants with conditions known to be associated with elevated cytokine levels and processed serum AMH samples immediately after collection without prolonged storage under suboptimal conditions.

There are other reasons for the differences in AMH levels, indicating clomiphene resistance, in the literature. For example, one study concluded that PCOS patients who were obese and had hyperandrogenemia were more likely to be resistant to CC therapy; they also found that AMH levels were even higher in this group of women [15]. Therefore, in CC resistant or failure cases with high AMH levels, standardization of AMH levels according to BMI and androgen elevation may more accurately indicate CC responsiveness. Our AMH results were unaffected by these conditions and were close to the reliable values. Therefore, an AMH level above 4.5 ng/mL may suggest the need to consider alternative ovulation-inducing strategies rather than CC as higher AMH levels appear to be associated with reduced ovulatory response in women with PCOS.

Although AMH elevation in women with PCOS affects the response to CC, this resistance does not affect Gn treatment because endogenous FSH secreted by the pituitary gland is insufficient to select more follicles when using CC [16,17]. To explain this insufficiency, a study analyzed 193 cases diagnosed with PCOS and found that the homozygous Ser/Ser polymorphism of FSH receptors was resistant to CC. In addition, these researchers claimed that, to overcome this high FSH threshold, higher basal FSH levels are required for ovulation [18]. However, another study found no evidence of a link between variants in the FSH receptor gene and AMH levels [19]. AMH may also inhibit the response of granulosa cells to FSH as an indicator of the number of small antral follicles in PCOS [19]. Although the number of studies performed to explain the relationship between FSH receptor problems and AMH is insufficient in cases that inhibit FSH, increasing the dose of Gns will decrease resistance to and inhibition of FSH and decrease FSH and AMH levels, as this causes ovulation [20]. Therefore, we claim that the predictive role of AMH in determining the response to OI with CC and Gn is significantly different in PCOS patients.

The follicular response to Gn treatment may vary according to AMH levels. High AMH levels play a crucial role in predicting the response to Gn treatment during ovarian stimulation in infertile women without PCOS [21]. However, in women with PCOS, the results have been conflicting. One study evaluated the effect of AMH on the outcome of ovarian stimulation in PCOS and found that circulating AMH levels were negatively correlated with the ovarian response to human menopausal gonadotropins [22]. However, another study found that AMH levels were significantly higher in responders to CC treatment than in non-responders [11]. Therefore, optimal serum AMH levels may be required for successful ovarian stimulation with different infertility etiologies.

Although the sensitivity and specificity of the identified cutoff were moderate, AMH should not be interpreted as a standalone predictor. Rather, it should be incorporated into a comprehensive clinical assessment including BMI, hyperandrogenism, ovarian morphology, and previous treatment response. From a clinical perspective, identifying women with elevated AMH levels prior to treatment initiation may help clinicians avoid prolonged and potentially ineffective CC therapy, thereby reducing psychological stress and treatment delays.

Interestingly, we observed longer ovarian stimulation durations in the pregnant group, suggesting that extended stimulation may partially overcome follicular resistance in selected patients. This finding warrants further investigation.

The limitations of this study include its retrospective design, relatively small sample size, inclusion of both CC-resistant and CC-failure patients in a single analysis, and the patients with a history of infertility were followed for a limited duration of approximately one year. Furthermore, other relevant outcome measures, including clinical pregnancy and live birth rates, were not assessed during the follow-up period. Serum androgen profiles were also unavailable for all participants, as hyperandrogenism was diagnosed on a purely clinical basis in some women. Despite these limitations, our results provide clinically relevant evidence supporting the role of AMH as a useful adjunctive marker in treatment planning for infertile PCOS women with CC resistance or failure.

## 5. Conclusions

In women with PCOS who demonstrate CC resistance or treatment failure, elevated serum AMH levels may be associated with lower IUI success rates. Assessment of AMH prior to OI could potentially help identify patients who are less likely to achieve pregnancy with conventional CC-based approaches, thereby supporting earlier consideration of alternative stimulation strategies. Nevertheless, further prospective, large-scale studies are required to establish standardized AMH cut-off values and to better define its role in predicting treatment outcomes in PCOS-related infertility.

## Figures and Tables

**Table 1:** Comparison of Demographic and Clinical Characteristics of Clomiphene Citrate Resistant or Unresponsive Patients According to Pregnancy Status.

Variables		Non-Pregnant (*n* = 57)	Pregnant (*n* = 36)	*p*-Value
Age (yr) *		25.9 ± 3.7	25.5 ± 4.7	0.671
BMI (kg/m^2^) *		24.3 ± 4.6	23.2 ± 3.1	0.310
Gravidity **		0 (0–1)	0 (0–1)	0.253
Parity **		0 (0–1)	0 (0–1)	0.742
Abortion **		0 (0–1)	0 (0–1)	0.066
Primary infertility ***		53 (91.2)	31 (86.1)	0.438
Infertility duration (yr) *		3.5 ± 2.0	2.7 ± 1.0	0.008
Current treatment ***				
	CC + IUI	25 (43.9)	17 (47.2)	0.892
	Gn + IUI	25 (43.9)	14 (38.9)	
	CC + Gn + IUI	7 (12.2)	5 (13.9)	
E2 (ng/mL) *		41.4± 18.5	42.5± 19.2	0.748
FSH (IU/L) *		6.8 ± 1.8	6.8 ± 1.7	0.931
LH (IU/L) *		7.8 ± 5.0	7.5 ± 4.4	0.714
Prolactin (IU/L) *		13.5 ± 5.9	12.3 ± 5.0	0.299
TSH (IU/L) *		2.1 ± 0.8	2.4 ± 1.3	0.163
Stimulation duration (days) *		13.7 ± 3.1	14.7 ± 3.3	0.028
Endometrium (mm) *		8.3 ± 2.2	8.6 ± 2.0	0.488
CC resistance ***		6 (10.5)	4 (11.1)	1.000
CC starting dose (mg) **		100 (100–150)	100 (100–150)	0.413
Gn starting dose (IU) **		37.5 (37.5–75)	37.5 (37.5–75)	0.292
Gn total dose (IU) *		563 ± 426	579 ± 179	0.475
Follicle size (mm) *		17.6 ± 0.8	17.8 ± 0.9	0.580
AMH (ng/mL) *		6.4 ± 6.3	4.1 ± 3.0	0.026

BMI: Body Mass Index; CC: Clomiphene Citrate; IUI: Intrauterine Insemination; Gn: Gonadotropin; E2: Estradiol; FSH: Follicle-Stimulating Hormone; LH: Luteinizing Hormone; TSH: Thyroid-Stimulating Hormone; AMH: Anti-Müllerian Hormone. * Data are presented as mean (±) standard deviation, Student’s *t*-test. ** Data are presented as median (minimum/maximum), Mann–Whitney U test. *** Data are presented as number (percentage). Chi-square or Fischer’s exact test. A *p* value < 0.05 is statistically significant.

**Table 2:** Multivariate Logistic Regression Analysis of Baseline Risk Factors Predicting Pregnancy in Women with PCOS and Prior Clomiphene Citrate Unresponsiveness.

Variable	B	S.E.	Wald	df	Sig.	Exp(B)	95% CI (Lower)	95% CI (Upper)
Age	−0.044	0.123	0.128	1	0.720	0.957	0.751	1.218
BMI	−0.014	0.099	0.020	1	0.889	0.986	0.813	1.197
AMH	0.140	0.054	6.623	1	0.010	1.151	1.034	1.280
Infertility duration	0.132	0.202	0.424	1	0.515	1.141	0.768	1.695
Constant	−2.254	4.010	0.316	1	0.574	0.105	—	—

BMI: Body Mass Index; AMH: Anti-Müllerian Hormone. A *p* value < 0.05 is statistically significant.

## Data Availability

Data can be requested from the authors upon reasonable request.

## Abbreviations

AMH	anti-Müllerian hormone
PCOS	polycystic ovary syndrome
OI	ovulation induction
CC	clomiphene citrate
IUI	intrauterine insemination
Gn	gonadotropin
E_2_	estradiol
FSH	follicle-stimulating hormone
LH	luteinizing hormone
TSH	thyroid-stimulating hormone
